# Influence of learning effect on reliability parameters and global indices of standard automated perimetry in cases of primary open angle glaucoma

**Published:** 2018

**Authors:** Uma Sharan Tiwari, Ankita Aishwarya, Akanksha Bhale

**Affiliations:** * Department of Ophthalmology, Gajra Raja Medical College, Gwalior, India

**Keywords:** standard automated perimetry, learning effect in perimetry, reliability parameters

## Abstract

**Aim.** To find out the influence of learning effect on various reliability parameters and global indices of standard automated perimetry in cases of primary open angle glaucoma.

**Method.** Thirty eyes of 30 patients of Primary Open Angle Glaucoma constituted material for this prospective observational study. All the patients underwent standard automated perimetry three times on three different days within one week on G program of Octopus Visual Field Analyzer. The details of reliability indices, global indices, and test duration were compiled and analyzed.

**Result.** A male preponderance was observed with a mean age of 53.43 years. The mean false positive was found to be 11.13, 6.07, and 2.40 on visit 1, visit 2, and visit 3, respectively. The mean false negative was found to be 13.73, 9.33, and 6.30 on visit 1, visit 2, and visit 3, respectively. The mean fixation loss was found to be 7.53, 3.83, and 1.60 on visit 1, visit 2, and visit 3, respectively. The mean deviation was found to be -10.14, -5.75, and -3.11 on visit 1, visit 2, and visit 3, respectively. The pattern standard deviation was found to be 5.58, 4.63, and 4.35 on visit 1, visit 2, and visit 3, respectively. The mean test duration was found to be 7.68, 6.21, & 5.51 minutes for visit 1, visit 2 and visit 3, respectively. All the values were statistically highly significant except for the pattern standard deviation.

**Conclusion.** At least three standard automated perimetry tests should be performed for all glaucoma patients in order to obtain a base line perimetry chart.

## Introduction

Factors that can influence results of standard automated perimetry (SAP) are technician’s experience, time of day, season etc. [**1**]. Reliability indices i.e. subject response indices are used to estimate how closely a patient complies with the instruction provided [**2**]. Fatigue and anxiety can negatively interfere with the test results [**3**]. Perimetry is a psychophysical test that requires patient’s co-operation and concentration. With repeated attempts, patient’s performance improves and this phenomenon is called “learning effect”. 

Hence, this study was conducted to find out the influence of learning effect on reliability parameters and global indices of SAP in cases of primary open angle glaucoma (POAG).

## Material & Method 

This prospective observational study was carried out on POAG patients attending the Glaucoma Clinic of our institution. Approval was obtained from the Institutional Ethical Committee prior to the study.

Thirty eyes of 30 patients of POAG constituted material for the present study. The following cases were excluded: 

1. Patients not willing to participate in the study. 

2. Patients having best corrected visual acuity < 6/ 60. 

3. Patients having retinal pathology, e.g. diabetic retinopathy, venous occlusion, etc. 

The SAP was performed by G program of Octopus Visual Field Analyzer on one eye of selected primary open angle glaucoma patients. All the patients underwent SAP three times on three different days within one week. The patient’s name, age, gender, date, time of the test, type of test performed, details of reliability indices, global indices, and test duration were recorded. 

The data obtained this way were compiled and analyzed with special reference to false positive (FP), false negative (FN), fixation loss (FL), the mean deviation (MD) and the pattern standard deviation (PSD). The mean, standard deviation, and P values calculated.

## Results

Out of 30 patients, 21 were males and 9 were females. The mean age was 53.43 years.

The mean false positive was found to be 11.13, 6.07, and 2.40 on visit 1, visit 2, and visit 3, respectively (**Table 1**). The mean false negative was found to be 13.73, 9.33, and 6.30 on visit 1, visit 2, and visit 3, respectively (**Table 2**). The mean fixation loss was found to be 7.53, 3.83, and 1.60 on visit 1, visit 2, and visit 3, respectively (**Table 3**). The mean deviation was found to be – 10.14, -5.75, and -3.11 on visit 1, visit 2 and visit 3, respectively (**Table 4**). The pattern standard deviation was found to be 5.58, 4.63, and 4.35 on visit 1, visit 2, and visit 3, respectively (**Table 5**). The mean test duration was found to be 7.68, 6.21 & 5.51 minutes for visit 1, visit 2, and visit 3, respectively (**Table 6**). All the values were statistically highly significant except for the pattern standard deviation. The PSD did not change significantly during second and third visits.

**Table 1:** False positive on three visits

Range of false positive %	Number of patients on different visits					
	Visit 1		Visit 2		Visit 3	
	No.	%	No.	%	No.	%
0-10	19	63	26	87	28	93
11-20	8	27	2	7	2	7
21-30	2	7	2	7	0	0
31 and above	1	3	0	0	0	0
Total	30	100	30	100	30	100
Mean	11.13		6.07		2.40	
Standard deviation		5.39		4.56		2.55
P value between visit 1 and 2 = 0.00023; P value between visit 2 and 3 = 0.00037						

**Table 2:** False negative on three visits

Range of false negative %	Number of patients on different visits					
	Visit 1		Visit 2		Visit 3	
	No.	%	No.	%	No.	%
0-10	8	27	26	87	28	93
11-20	19	63	2	7	2	7
21-30	3	10	2	7	0	0
31 and above	0	0	0	0	0	0
Total	30	100	30	100	30	100
Mean	13.73		9.33		6.30	
Standard deviation		5.36		3.52		2.17
P value between visit 1and 2 =0.0004; P value between visit 2 and 3 =0.0002						

**Table 3:** Fixation loss on three visits

Range of fixation loss %	Number of patients on different visits					
	Visit 1		Visit 2		Visit 3	
	No.	%	No.	%	No.	%
0-10	25	83	27	90	29	97
11-20	2	7	1	3	1	3
21-30	2	7	2	7	0	0
31 and above	1	3	0	0	0	0
Total	30	100	30	100	30	100
Mean	7.53		3.83		1.60	
Standard deviation		6.17		5.36		2.01
P value between visit 1 and 2 = 0.0161; P value between visit 2 and 3 = 0.0391						

**Table 4:** Mean deviation on three visits

Range of mean deviation (negative)	Number of patients on different visits					
	Visit 1		Visit 2		Visit 3	
	No.	%	No.	%	No.	%
0-6	11	37	20	67	26	87
7-14	10	33	9	30	4	13
15-21	2	7	1	3	0	0
21 and above	7	23	0	0	0	0
Total	30	100	30	100	30	100
Mean	-10.14		-5.75		-3.11	
Standard deviation		8.97		5.17		2.96
P value between visit 1 and 2 = 0.0247; P value between visit 2 and 3 = 0.0191						

**Table 5:** Pattern Standard deviation on three visits

Range of Pattern Standard deviation	Number of patients on different visits					
	Visit 1		Visit 2		Visit 3	
	No.	%	No.	%	No.	%
0-3	6	20	10	33	30	100
4-6	10	33	12	40	0	0
7-9	10	33	8	27	0	0
10 and above	4	13	0	0	0	0
Total	30	100	30	100	30	100
Mean	5.58		4.63		4.35	
Standard deviation		2.84		2.13		2.05
P value between visit 1and 2 = 0.151; P value between visit 2 and 3 = 0.601						

**Table 6:** Time duration to perform test on three visits

Duration(minutes)	Number of patients on different visits					
	Visit 1		Visit 2		Visit 3	
	No.	%	No.	%	No.	%
0-5	5	17	10	33	17	57
5-10	19	63	16	53	11	37
10-15	6	20	4	13	2	7
15 and above	0	0	0	0	0	0
Total	30	100	30	100	30	100
Mean	7.68		6.21		5.51	
Standard deviation		2.80		2.28		2.03
P value between visit 1and 2 = 0.029; P value between visit 2 and 3 = 0.212						

## Discussion

Visual field testing is crucial not only in diagnosis but also in assessing the progression of glaucoma. However, obtaining a good baseline visual field may not be easy because visual field testing can be influenced by a number of factors such as education level of patient, unaccustomed environment, skill of technician, stage of disease, concentration, cooperation, and motivation of patient. As a result, the first field may not be reliable. On repeat field examination, after explaining the procedure to patient, the subsequent fields may be more reliable. A good baseline field is one that has reliability parameters within acceptable limits. 

To make SAP reliable, one needs full cooperation and comfortable seating of the patient, better understanding of the test and accurate setting of the parameters of the machine so that there is less chance of false positive and false negative catch trials and reliability factor remains within normal limits [**4**]. The greater rejection rate among glaucomatous subjects was found to be due to their higher rate of fixation loss and false-negative responses [**5**]. Studies revealed that perimetry results are significantly influenced by the level of experience in patients, which is gained during first session of test performed, which further leads to a better result when the test is performed second and third times [**6**,**7**]. For a good reliable result on standard automated perimetry, at least three tests should be performed in an individual before any conclusion [**8**,**9**]. 

In present series, a male preponderance was observed and the mean age was found to be 53.43 years, which is in accordance with demographic characteristics of POAG; other workers made similar observations [**4**]. 

It was observed that when patients were tested twice or thrice by perimtery there was significant improvement in reliability indices, MD, PSD and test duration. Other workers [**8**] also observed that there was a learning curve which could be the psychological phenomenon of the visual system adapting to the process, or it could be the psychological effect that influences a patient’s decision-making as to whether they saw a stimulus or not. There is significant learning and fatigue effects in perimetry. Therefore, for good and reliable results on perimetry, at least 3 tests should be performed in an individual. Between the tests of both eyes, a rest should be offered. 

We observed that out of all reliability indices, fixation loss was a common cause of poor results in glaucoma subjects. High false negative was another reason for unreliable fields. These findings are comparable with the findings of other workers [**5**]. In most of the patients, this could be due to patient’s fatigue or distraction and failure to understand the test [**3**].

It was observed that on repeated testing, MD showed improvement but PSD remained unaffected. 

This may be because PSD represents damage specifically produced by glaucoma and is reproducible. Other workers [**1**] also made similar observations. It was observed that the test duration decreases with second and third tests and improves the reliability indices. Longer duration of test may result into exhaustion. With repeated testing, the patient learns to perform it quickly which results in shorter test duration and consequently less fatigue and better test results. 

There are many factors affecting the reliability of visual field examination in glaucoma patients, which depends on the instructions given to patients, patient’s cooperation, comfortable seating of patients, and better understanding of test by patient, fatigue, distraction, anxiety during test and accurate setting of parameters of machine [**4**,**9**]. If all these factors are considered prior to the commencement of test and taken care of, the learning curve can be small and better results can be obtained. It is understood that the first and second visual field may not be reliable due to the learning curve effect. Only the third visual field should be used for diagnosis and follow-up analysis. 

Hence, it can be concluded that at least three SAP tests should be performed for all glaucoma patients in order to obtain a base line perimetry chart.

**Financial support and disclosure**

None.

## References

[1] Franciso G, Montolio J, Wasselink C, Gordinj M, Jansonius NM (2012). Factors that influence SAP test results in glaucoma. Investigative Ophthalmology & Visual Science.

[2] IPS standards and guidelines 2010.

[3] Macedo Batista Fiorelli V, Kasahara N, Cohen R, Santucci França A, Della Paolera M, Mandia C Jr., Vicente de Almeida G (2006). Improved automated perimetry performance following exposure to Mozart. Br J Ophthalmol.

[4] Uzma F, Arshad S, Nisar S, Fehmi MS, Jafri R, Atiya R (2009). Evaluation of reliability of visual field examination in glaucoma patients. Pak J Ophthalmol.

[5] Katz J, Quigley HA, Sommer A (1995). Repeatability of the Glaucoma Hemifield Test in Automated Perimetry. Investigative Ophthalmology & Visual Science.

[6] Heijl A, Lindgren G, Olsson J (1989). Effect of perimetric experience on normal individual. Arch Ophthalmol.

[7] Castro DP, Kawase J, Luiz Alberto (2008). Study on learning effect of standard automated perimetry experience in normal individuals. Arq Bras Oftalmol.

[8] Lamparter J, Schulze A, Schuff AC, Berres M, Pfeiffer N, Hoffmann EM (2011). A cross sectional study to see the learning curve and fatigue effect. Am J Ophthalmol.

[9] Kutzko KE, Brito CF, Wall M (2000). A study to see the effect of perimetrist instruction on conventional perimetry. Invest. Ophthalmol. Vis. Sci.

